# Going beyond randomised controlled trials to assess treatment effect heterogeneity across target populations

**DOI:** 10.1002/hec.4903

**Published:** 2024-09-26

**Authors:** David G. Lugo‐Palacios, Patrick Bidulka, Stephen O’Neill, Orlagh Carroll, Anirban Basu, Amanda Adler, Karla DíazOrdaz, Andrew Briggs, Richard Grieve

**Affiliations:** ^1^ Department of Health Services Research and Policy London School of Hygiene & Tropical Medicine London UK; ^2^ Department of Non‐Communicable Disease Epidemiology London School of Hygiene & Tropical Medicine London UK; ^3^ The Comparative Health Outcomes, Policy & Economics (CHOICE) Institute University of Washington School of Pharmacy Seattle Washington USA; ^4^ Diabetes Trials Unit The Oxford Centre for Diabetes, Endocrinology and Metabolism University of Oxford OCDEM Building Churchill Hospital Headington UK; ^5^ Department of Statistical Science University College London London UK

**Keywords:** diabetes mellitus, electronic health records, heterogeneous treatment effects, instrumental variables, target trial emulation, transportability

## Abstract

Methods have been developed for transporting evidence from randomised controlled trials (RCTs) to target populations. However, these approaches allow only for differences in characteristics observed in the RCT and real‐world data (overt heterogeneity). These approaches do not recognise heterogeneity of treatment effects (HTE) according to unmeasured characteristics (essential heterogeneity). We use a target trial design and apply a local instrumental variable (LIV) approach to electronic health records from the Clinical Practice Research Datalink, and examine both forms of heterogeneity in assessing the comparative effectiveness of two second‐line treatments for type 2 diabetes mellitus. We first estimate individualised estimates of HTE across the entire target population defined by applying eligibility criteria from national guidelines (*n* = 13,240) within an overall target trial framework. We define a subpopulation who meet a published RCT's eligibility criteria (‘RCT‐eligible’, *n* = 6497), and a subpopulation who do not (‘RCT‐ineligible’, *n* = 6743). We compare average treatment effects for pre‐specified subgroups within the RCT‐eligible subpopulation, the RCT‐ineligible subpopulation, and within the overall target population. We find differences across these subpopulations in the magnitude of subgroup‐level treatment effects, but that the direction of estimated effects is stable. Our results highlight that LIV methods can provide useful evidence about treatment effect heterogeneity including for those subpopulations excluded from RCTs.

## 1 INTRODUCTION

Health policy‐makers and reimbursement agencies require evidence of comparative effectiveness for target populations and subpopulations relevant to the decision context. Randomised controlled trials (RCTs) are the recommended source of evidence for estimating treatment effects (ICER, 2020; NICE, 2013). However, the selection of trial centres and participants leads to differences in the characteristics of trial populations versus those eligible for the same interventions in routine practice, that is, the ‘target populations’. RCT eligibility criteria, especially for trials designed to assess safety and efficacy rather than comparative effectiveness, may exclude subpopulations of interest to national and local decision‐makers, with these exclusions partly captured by observed measures (Dahabreh & Hernán, 2019; Elliott et al., 2023; Gheorghe et al., 2013; Hartman et al., 2015; Stuart et al., 2010). Statistical methods have been developed to ‘transport’ estimates of average treatment effects (ATE) or conditional average treatment effects (CATEs) from RCTs to target populations and subpopulations defined by routine data (Allcott & Mullainathan, 2012; Dahabreh & Hernán, 2019; Degtiar & Rose, 2023; Elliott et al., 2023; Hartman et al., 2015; Stuart et al., 2010). Notably (Henderson et al., 2017; Varadhan et al., 2016), propose a cross‐design synthesis approach that leverages patient‐level RCT and observational data to account for confounding in the observational data, and allows for heterogeneity of treatment effects (HTE) according to baseline prognostic measures such as individuals' baseline risk. However, these methods only account for HTE according to observed characteristics (overt heterogeneity). RCT participation may also reflect characteristics that are *unmeasured* in both the RCT or routine data, such as frailty levels, attitude to risk, preferences, or contextual factors such as quality of care. If unmeasured characteristics associated with RCT participation also modify the relative effectiveness of the treatment alternatives, that is, there is essential heterogeneity, then this leads to *study selection bias and* related concerns about the transportability of the results to the decision context (Hartman et al., 2015).

Non‐randomised studies (NRS) can provide complementary evidence on comparative effectiveness in subpopulations excluded from RCTs and may be less prone to sample selection bias (Imai et al., 2008). The improved quality and availability of data from electronic health records (EHR) (EHRs) offer new opportunities for NRS to provide estimates of treatment effects including for subgroups who do not meet explicit measured RCT eligibility criteria. A further advantage is that an NRS may include the treatment comparators of direct interest to the decision‐context and incorporate treatment protocols and adherence levels from routine practice rather than those driven by the RCT design. However, the major challenge when using NRS to assess comparative effectiveness is that the treatments groups of interest are selected according to measured and unmeasured prognostic measures which leads to *treatment selection bias* (confounding). A recommended NRS design to reduce the risk of confounding and other sources of bias is ‘target trial emulation’ (Gomes et al., 2022; Hernán et al., 2016; Hernán and Robins, 2016; Moler‐Zapata et al., 2023b; NICE, 2021; Wang et al., 2023). The target trial framework requires the analyst to conceptualise the NRS as if it were an RCT, and make explicit decisions at the design stage to reduce the risk of bias, for example, by defining the study populations, treatment regimes, and analytical methods. The target trial framework has helped some NRS replicate treatment effect estimates from RCTs (Hernán and Robins, 2016; Wang et al., 2023), and the recent update to the NICE methods guide advocates its use when RCT evidence on comparative effectiveness is unavailable or is judged insufficient (NICE, 2021). However, if the target trial applies the same eligibility criteria as an RCT, it may face some of the same concerns about sample selection bias, especially as the stated eligibility criteria (e.g., age) are likely to be correlated with measures (e.g., frailty levels) unobserved in the data used by the target trial. A promising approach is to design a target trial to assess HTE across the full target populations and subpopulations of decision‐making relevance, including subpopulations who would have been ineligible for the published RCT(s). For such a target trial design to provide evidence on comparative effectiveness of direct relevance to decision‐makers, it is essential to reduce the risk of unobserved confounding, but also of essential heterogeneity across the subpopulations of interest.

Instrumental Variable (IV) methods, such as two‐stage least squares (2SLS), can reduce the risk of unobserved as well as observed confounding. However in settings with essential heterogeneity, 2SLS no longer identifies policy‐relevant estimands, such as the ATE, even if the instrument is strong and valid (Heckman et al., 2006). Local IV (LIV) approaches can provide consistent estimates of the ATE and CATEs (Cornelissen et al., 2016; Heckman & Vytlacil, 2005). LIV methods draw on choice theory to identify ‘marginal treatment effects’ (MTEs) for those at the ‘margin of treatment choice’ for whom the level of a continuous IV balances observed and unobserved characteristics (Björklund & Moffitt, 1987; Heckman & Vytlacil, 1999). For these patients at the ‘marginal choice’, a small change in the level of the valid continuous IV changes the treatment decision, without altering the distribution of the underlying risk factors. Therefore, we can identify MTEs for individuals who *comply* with the change in treatment that is due to a small change in the level of the IV, by comparing mean outcomes between two groups of similar patients who are only separated by a small change in the IV (Heckman & Vytlacil, 1999). Hence, given observed covariates and common support, MTEs can be estimated along the continuum of the IV and aggregated to provide CATEs and ATEs (Heckman and Vytlacil, 1999, 2001, 2005).

The theoretical properties of these LIV methods in settings with essential heterogeneity have been established by (Angrist & Fernández‐Val, 2013; Basu et al., 2007; Heckman et al., 2006) inter alia. A recent simulation study showed that given an IV of sufficient strength, an LIV method can provide consistent estimates of treatment effects in settings with overt and essential heterogeneity (Moler‐Zapata et al., 2023a). LIV methods have been applied across a range of settings including cardiovascular and bariatric surgery, universal child care programs, and transfers to intensive care units (Basu et al., 2018; Cornelissen et al., 2018; Grieve et al., 2019; Reynolds et al., 2021).

In this paper, we use a LIV method to examine HTE across an entire target population. We recognise that there may be essential heterogeneity for subpopulations in the target population who were ineligible for the RCT. We illustrate our approach for addressing this challenge within the running example of the PERsonalised Medicine for Intensification of Treatment (PERMIT) study which evaluates alternative second‐line drug treatments for people with type 2 diabetes mellitus (T2DM) treated with metformin (Bidulka et al., 2021). Here, we compare two second‐line treatments, dipeptidyl peptidase‐4 inhibitors (DPP4i) and sulfonylureas (SU) as ‘add on’ therapies to metformin (for details see (Bidulka et al., 2021)). The primary endpoint is improvement in glycaemic control, measured by the change in haemoglobin A1c (HbA_1c_) between baseline and 1‐year follow‐up. Published RCTs report divergent results, with some reporting that SUs lead to greater improvements in HbA_1c_ than DPP4i, and others that DPP4i lead to greater improvements (GRADE Study Research Group, 2022; Marathur et al., 2016; Rosenstock et al., 2019). A general problem is that the RCTs have explicitly excluded people with poor glycaemic control who are likely to differ in their responses to the treatments compared to people with better glycaemic control at baseline. Consequently, treatment recommendations based on RCT evidence may be suboptimal for subpopulations excluded from these studies. Faced with insufficient evidence from RCTs including the full target population of interest, NICE clinical guidelines have recommended allowing for individual's risk factors and circumstances when choosing second‐line treatments between options that include DPP4is and SUs, but there is little evidence on HTE to help decision‐makers transport the findings from RCTs to target populations of interest. Hence, the PERMIT study exemplifies the common situation where decision‐makers require further evidence on HTE including subgroups excluded from an RCT.

The paper proceeds as follows. In Section 2, we offer an overview of the main features of the PERMIT study. In Section 3, we outline the LIV approach for handling confounding and examining heterogeneity. In Section 4, we define target trial protocols for the overall target population of interest, and subpopulations who do and do not meet ‘RCT eligibility criteria’. In Section 5, we report the empirical results. In Section 6, we discuss the results in the context of the extant literature for examining HTE when transporting results from RCTs to the routine practice setting, and outline future research directions.

## 2 OVERVIEW OF THE PERMIT STUDY

The PERMIT study aims to assess the comparative effectiveness of alternative second‐line pharmacological treatments for people with T2DM in England who meet national eligibility criteria for these treatments, and to provide evidence to help personalise the choice of second‐line treatment according to individual‐level characteristics. In this paper, we evaluate DPP4i versus SU which were the most prevalent classes of second‐line treatments for T2DM in the UK between 2011 and 2015 (Wilkinson et al., 2018). We used Clinical Practice Research Datalink (CPRD) data from England on clinical and demographic characteristics, clinical diagnoses, laboratory test results, prescribing, and outcome information recorded in primary care, with further information on resource use and outcomes from linkage to hospital episodes statistics data (Herbert et al., 2017; Herrett et al., 2015; Wolf et al., 2019). Full details of the PERMIT study have been published elsewhere (Bidulka et al., 2021, 2023, 2024). In brief, we undertook a target trial emulation to define the target populations and treatment comparisons of interest from the CPRD data. The study addressed the potential concerns about confounding with a continuous preference‐based IV (Baiocchi et al., 2014). The IV was the clinical commissioning groups (CCG)'s tendency to prescribe (TTP) DPP4i as second‐line treatment. Over the study time‐frame, general practitioners (GPs) worked within a CCG which informed health funding decisions for its respective geographic region. For example, some CCGs tended to recommend –to their affiliated GPs– the prescription of either DPP4i or SU as second‐line treatment (Bidulka et al., 2023; Wilkinson et al., 2018). Hence, in keeping with these previous studies (Bidulka et al., 2023, Wilkinson et al., 2018), we defined the IV at the CCG level rather than at the individual GP level as this reflected the major influence of CCGs on decision making. A further advantage of defining the IV at a CCG‐level IV is that it is not influenced by unobserved individual‐level characteristics. However, this may come at the cost of reduced IV strength since there is typically less variation at the more aggregated level. To preserve variation and increase IV strength, we defined the IV for each patient by calculating the proportion of patients prescribed the treatment within a window of 365 days preceding that patients index date. For further details on the assumptions underlying preference‐based IVs see (Widding‐Havneraas et al., 2021).

**FIGURE 1:**
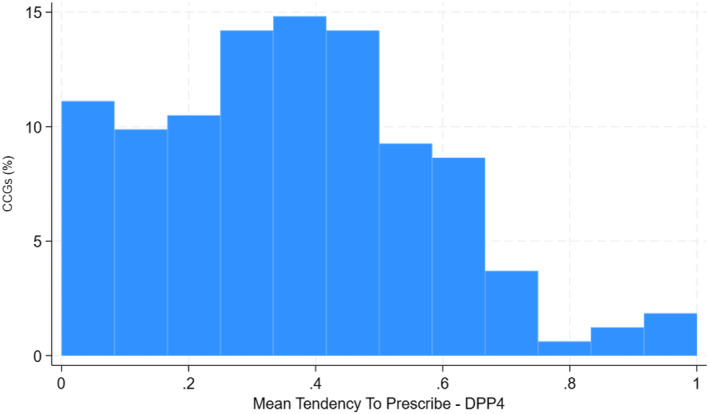
Variation in mean TTP‐DPP4i across 162 NHS CCGs in the one year prior to first‐line treatment intensification (2011–2015). DPP4i, dipeptidyl peptidase‐4 inhibitors; TTP, tendency to prescribe.

We exploited the wide variation across CCGs in the proportion of people prescribed DPP4is versus SUs (see Figure 1). This ‘natural variation’ implied that people of similar baseline prognosis received a different second‐line treatment simply according to their CCG. We defined TTP as the proportion of eligible people prescribed DPP4i second‐line treatment within the 12 months preceding the specific baseline (time zero) for each person. That is, while the IV is defined at the CCG level, it changes from patient to patient within the same CCG. A valid instrument must meet four main conditions (see also the Direct Acyclic Graph in Figure S1 of the online supporting material; Baiocchi et al., 2014). First, the instrument must predict the treatment prescribed (Staiger & Stock, 1997). Here, we assessed the relevance of the CCGs TTP with a weak instrument test that is robust to heteroscedasticity and clustering by NHS region, and reported F‐statistics of around 1115, compared to a benchmark of 100 (Baiocchi et al., 2014; Moler‐Zapata et al., 2023a). Second, the instrument must be independent of unmeasured covariates that predict the outcomes of interest, which can be partially evaluated through its relationship with measured covariates. We found that levels of observed prognostic covariates were similar across levels of the instrument (see Figure S2 of the online supporting material). Third, the instrument must have an effect on the outcomes only through the treatment received. We adjusted for contextual and temporal confounders, and made the weaker assumption, that the TTP was independent of the outcome, and only had an effect on the outcome via the treatment received, *after* adjusting for any differences in region, GP practice size, and time period (see Online Supplement). Fourth, we assume that the average treatment choice must increase or decrease monotonically with the level of the IV (Vytlacil, 2002). Here, it is plausible to assume that if a group of patients who attended a CCG with a moderate preference for prescribing DPP4is were prescribed this drug class, then a similar group of patients who attended a CCG with a stronger preference for prescribing DPP4is would also be prescribed this drug class.

The PERMIT study previously used the 2‐stage residual inclusion (2SRI) to report ATEs for the overall target population of interest (Bidulka et al., 2024). However, an outstanding concern is to explore HTE, that may arise according to baseline risk factors that are observed (e.g., HbA_1C_) as well as those that are not readily observed (e.g., patient preferences). We now formally state the IV assumptions and define the LIV approach for estimating policy relevant estimands of interest, namely ATE and CATEs.

## 3 METHODS

### 3.1 Instrumental variables methods

Following the Neyman‐Rubin potential outcomes framework (Rubin, 1974; Neyman, 1990), let *Y*_*D*_ denote the observed outcome, *D*_*Z*_ the choice of treatment, *Z* the IV observed for each individual (*Y*_*D*_,*D*_*Z*_,*Z*). Let *Y*_1_ = *μ*_1_(*X*_*O*_,*X*_*U*_,*ϑ*) and *Y*_0_ = *μ*_0_(*X*_*O*_,*X*_*U*_,*ϑ*) denote the individual's potential outcomes under each treatment, where *X*_*O*_ and *X*_*U*_ are vectors of measured and unmeasured confounders, and *ϑ* captures the remaining unobserved random variables which influence outcomes, but not treatment selection. We assume exogeneity of the covariates (A1), so that the treatment assignment is the only source of endogeneity, such that (*X*_*O*_,*X*_*U*_) ⊥ *ϑ* and *X*_*O*_ ⊥ *X*_*U*_.

#### 3.1.1 Identification assumptions

We follow Abadie (2003) and Tan (2006) in making the following assumptions which are the conditional version of the assumptions outlined by Angrist et al. (1996) for the local average treatment effect (LATE):

**Table:** 

(A2)	Unconfoundedness of *Z*	$\left(Y_{d_{z}},D_{z}\right)⊥Z|X_{O}$
(A3)	Exclusion restriction	$Y_{d_{z}}=Y_{d}$ with probability 1
(A4)	Relevance	0 < *P*(*Z* = *z*) < 1
(A5)	Monotonicity	If *z* ^′^ > *z* then $D_{z^{\prime }}\geq D_{z}$ with probability 1
(A6)	Stable unit treatment value assumption	*D* = *D*_*Z*_ and *Y* = *Y*_*D*_

Assumption (A2) requires that within levels of *X*_*O*_ *Z* is as good as randomly assigned. Assumption (A3) rules out that *Z* has a direct effect on the outcome other than through *D*_*z*_. Assumptions (A2) and (A3) ensure that the only effect of the *Z* on the outcome is through *D*_*z*_. Assumption (A4) ensures that *Z* and *D*_*z*_ are correlated conditional on *X*_*O*_. Assumption (A5) requires that an increase in *Z* always results in a higher or equal level of treatment assignment, and this is needed to point‐identify our estimand of choice. Assumption (A6) requires that one individual's potential outcomes (*Y*_*D*_) and treatments (*D*_*z*_) are not influenced by other individuals' levels of *Z* (i.e., no interference), nor by how the instrument or treatment is delivered (i.e., no different versions of *Z* or *D*_*z*_).

#### 3.1.2 Estimands

(Angrist et al., 1996; Imbens and Angrist, 1994) show that following the above assumptions, the LATE can be defined as ${∆}^{LATE}\left(x_{o},z,z^{\prime }\right)=E\left[Y_{1}-Y_{0}|X_{O}=x_{o},D_{z}<D_{z^{\prime }}\right]$ and is identified by the IV estimand:

$$\frac{E\left[Y|X_{O}=x_{o},Z=z^{\prime }\right]-E\left[Y|X_{O}=x_{o},Z=z\right]}{E\left[D|X_{O}=x_{o},Z=z^{\prime }\right]-E\left[D|X_{O}=x_{o},Z=z\right]}$$

(Tan, 2006; Vytlacil, 2002) showed that the independence (A2 and A3) and monotonicity assumptions (A5) within the LATE framework are equivalent to those imposed by a non‐parametric selection model, where treatment assignment depends on whether a latent index (*μ*_*D*_(*X*_*O*_,*Z*)) crosses a particular threshold ($X_{U_{D}}$):

$$D_{z}=1\left\{\mu_{D}\left(X_{O},Z\right)\geq X_{U_{D}}\right\}$$

where $X_{U_{D}}$ is a random variable that captures *X*_*U*_ and all other factors influencing treatment assignment but not the outcomes. We follow (Heckman and Vytlacil, 1999, 2001), in rewriting this equation as *D*_*z*_ = 1{*P*(*X*_*O*_,*Z*) > *V*}, where $V=F_{X_{U_{D}}}\left[X_{U_{D}}|X_{O}=x_{O},Z=z\right]$ with *V* ⊥ (*Z*,*X*_*O*_) and $P\left(x_{O},z\right)=F_{X_{U_{D}}|x_{O},z}\left[\mu_{D}\left(X_{O},Z\right)\right]$ is the propensity for treatment, and *F* represents a cumulative distribution function. *V* reflects the degree to which unmeasured confounders discourage the receipt of DPP4i (i.e., the effect of unobservables on the propensity of not receiving DPP4i). Therefore, for any arbitrary distribution of $X_{U_{D}}$ conditional on *X*_*O*_ and *Z*, by definition *V* –Uniform[0,1] conditional on *X*_*O*_ and *Z*. Then, the MTE can be defined as, ∆^MTE^(*x*_*O*_,*p*) = *E*(*Y*_1_ − *Y*_0_|*X*_*O*_ = *x*_*O*_,*V* = *v*) and (Heckman and Vytlacil, 1999, 2001) showed that, under the standard IV assumptions, it can be identified by:

$$\frac{\partial E_{\vartheta }\left(Y|X_{O}=x_{o},Z=z\right)}{\partial p}=E_{\vartheta }\left[\left(Y_{1}-Y_{0}\right)|X_{O}=x_{o},V=v\right]=\text{MTE}\left(x_{O},z\right)$$

where *Y* = *D*_*z*_ × *Y*_1_ + (1 − *D*_*z*_) × *Y*_0_ is the observed outcome, *v* = *P*(*x*_*O*_,*z*) and *p* is the propensity score.

(Heckman et al., 2006) showed that MTEs can be aggregated to obtain estimates of the ATE. (Basu, 2014) showed that MTEs can be used to derive personalised treatment (PeT) effects for each individual that recognise the plausible range of values that *V* may take for each patient, compatible with the levels of their observed covariates, the IV and their observed treatment assignment (see next section). The crucial insight underlying this approach is that given the observed covariates and the level of the IV, the treatment assignment status observed provides some information on $X_{U_{D}}$. For patients in the treatment group (*D*_*z*_ = 1), the propensity to choose treatment based on *X*_*O*_ and *Z* must outweigh the propensity to choose the comparator strategy based on $X_{U_{D}}$, that is, *P*(*x*_*O*_,*z*) > *v*. For patients in the comparator strategy (*D*_*z*_ = 0), the converse is true. The PeT effect for an individual is therefore obtained by averaging the MTEs corresponding to that individual's level of *X*_*O*_ and *Z* over levels of unobserved variables that are compatible with the individual's assigned treatment. Hence, ∆^PeT^(*x*_*O*_,*p*,*D*) = *E*(*Y*_1_ − *Y*_0_|*X*_*O*_ = *x*_*O*_,*P*(*z*,*x*_*O*_) > *v*) for individuals with *D*_*z*_ = 1 and ∆^PeT^(*x*_*O*_,*p*,*D*) = *E*(*Y*_1_ − *Y*_0_|*X*_*O*_ = *x*_*O*_,*P*(*z*,*x*_*O*_) < *v*) for individuals with *D*_*z*_ = 0.

All required treatment effect estimands, including ATE and CATEs, can be derived by appropriately aggregating the PeT effects since these are defined at the individual level (see next section).

### 3.2 Local instrumental variable estimator: Estimating PeT effects

(Basu, 2014, 2015) provides a detailed description for using LIV methods to estimate PeT effects. Briefly, the analyst must first estimate the propensity for treatment *p*(*x*_*O*_,*z*), based on observed covariates and the instrument. Next, the outcome *Y* is regressed on *X*_*O*_ and a function of $\hat{p}\left(x_{O},z\right)$ including interactions with *X*_*O*_. The approach outlined in (Basu, 2014) involves differentiating the outcome model *g*(*Y*) by $\hat{p}\left(x_{O},z\right)$ to obtain MTE estimates. Next, PeT effects for each individual can be obtained by performing numerical integration, with MTE $\left(\partial \hat{g}\left(Y\right)/\partial \hat{p}\right)$ evaluated by replacing $\hat{p}$ using 1000 random draws of $u∼\text{unif}\left(\min\left(\hat{p}\left(x_{O},z\right)\right),\max\left(\hat{p}\left(x_{O},z\right)\right)\right)$. Then, the corresponding treatment assignment at each value of *u* is given by $D^{*}=\Phi^{-1}\left\{\hat{p}\left(x_{O},z\right)\right\}+\Phi^{-1}\left(1-u\right)$. PeT effects can be computed by averaging $\partial \hat{g}\left(Y\right)/\partial \hat{p}$ over values of *u* consistent with the observed treatment decision (*D* ^∗^ > 0 if *D*_*z*_ = 1; or over values of *D* ^∗^ ≤ 0 if *D*_*z*_ = 0). Finally, an estimate of the ATE for the population can be obtained by averaging PeT effects over all of the observations, and the CATE for the subgroups of interest by aggregating over the appropriate strata of *X*_*O*_. Standard errors can be computed using nonparametric bootstrap methods (Basu, 2015).

## 4 TARGET TRIAL DESIGN

### 4.1 Overview

The aim of the target trial was to emulate a hypothetical RCT for estimating the ATEs and CATEs of second‐line treatment with DPP4i versus SUs in routine clinical practice in England. The LIV approach was applied to estimate individual‐level treatment effects which were aggregated across the entire target population to report ATEs, and for a small number of pre‐specified subgroups to report CATEs. We identified subpopulations from within the target population who met a published RCT's eligibility criteria (‘RCT eligible’) and a subpopulation who did not (‘RCT ineligible’). We compared the ATE for the ‘RCT eligible’ to the RCT with the same eligibility criteria (the ‘RCT benchmark’). We then compared CATEs for the overall target population, and the ‘RCT eligible’ and ‘RCT ineligible’ subpopulations. The CATEs were according to pre‐specified subgroups for whom there were prior hypotheses of HTE for DPP4i versus SUs. These subgroups were: age group (younger than 50 years old, 50–59, 60–69. 70–78, 79 or over; Khunti et al., 2018; Nauck et al., 2007), ethnicity (white, Asian (South Asian), black/mixed/other; Gan et al., 2020), baseline levels of HbA_1C_ measured in mmol/mol (<44; 44 to <64; 64 to <75; 75 to < 88; ≥88; Canivell et al., 2019; Nauck et al., 2007) and body mass index (BMI) defined according to WHO categories (Khunti et al., 2018).

### 4.2 Eligibility criteria for identifying the overall target population

We used the eligibility criteria stipulated in NICE clinical guidelines for T2DM to define the overall target population (NICE, 2022). Individuals had to initiate second‐line antidiabetic treatment with either SU or DPP4i in addition to metformin between January 1, 2011 and December 31, 2015 (for specific inclusion criteria see Table S1 in the online supporting material). These eligibility criteria ensured the target population was of direct relevance to the decision problem, and that there was general equipoise in the choice of SU and DPP4i, as either second‐line treatment was an option in the target population irrespective of their baseline characteristics.

### 4.3 Treatment strategies and ‘day zero’

The treatment strategies were SU or DPP4i for second‐line treatment with each drug class prescribed as an addition to metformin monotherapy. The definition of the comparators was broad to allow for any drug within either drug class, and included the specific drugs defined by the randomised groups in published RCTs (GRADE Study Research Group, 2022; Nauck et al., 2007; Rosenstock et al., 2019). *Day zero* was analogous to the time of randomisation and was when an individual met all eligibility criteria, had a first prescription for either SUs or DPP4is, and therefore started follow‐up.

### 4.4 Covariates

We pre‐specified baseline covariates for consideration in the LIV and alternative analyses and to define pre‐specified subgroups for estimating CATEs. From the CPRD‐HES linked data, we defined patient sociodemographic characteristics; for example, age, sex, ethnicity (see Table S2 for full list and definitions). We also extracted information on HbA_1c_, systolic blood pressure (SBP), diastolic blood pressure (DBP), estimated glomerular filtration rate (eGFR), and BMI using the most recent measures recorded in primary care. For HbA_1c_ we only considered the most recent measure within 180 days prior to baseline, and for SBP, DBP, and eGFR, we used the most recent measure within 540 days prior to baseline (Wilkinson et al., 2018).

### 4.5 Outcome and causal contrast of interest

The primary outcome was the change in HbA_1c_ between day zero and week 52 reported in mmol/mol. We reported the difference in the change as ∆ DPP4i ‐ ∆ SU, such that a negative difference meant that DPP4i were better (reduced HbA_1c_ more), and a positive difference that SUs were better. A clinically meaningful between‐treatment difference in HbA_1c_ was defined as 3.3 mmol/mol (0.3%) (European Medicines Agency, 2018). We used the HbA_1c_ measurement closest in time to the 1‐year follow‐up timepoint and allowed for measures within ±90 days, otherwise the measure was designated as missing (see Statistical Analysis section).

The causal contrast of interest was the ‘intention‐to‐treat’ (ITT) effect of DPP4i versus SU and so the full sample who met the eligibility criteria were considered in the analysis including those who switched to ‘third‐line’ treatments, or did not adhere to their ‘assigned’ treatment within the 12‐month follow‐up (see also statistical analysis, missing data).

### 4.6 Selection of RCT benchmark

We reviewed the literature to identify published RCTs that evaluated second‐line antidiabetic drugs as add‐on treatments to metformin for people with T2DM. Our selection criteria for emulating and benchmarking purposes were that the RCT: had to randomise between SUs and DPP4i, and report essential details required for emulation, including the eligibility criteria, treatment strategies and primary outcome (see Hanlon et al., 2024 and Supporting Note S1). We selected the RCT by (Nauck et al., 2007) as it was the only trial that met all the inclusion criteria. The Nauck et al. trial was a multinational, parallel‐group RCT with a non‐inferiority design that compared the efficacy of DPP4is versus SUs in patients with T2DM and inadequate glycaemic control following metformin monotherapy. The primary analysis of this RCT assessed whether DPP4is were non‐inferior versus SUs with regard to the change in HbA_1c_ from baseline to Week 52 according to a non‐inferiority margin of 3.3 mmol/mol (0.3%). The RCT exemplified key challenges in the use of RCTs designed to evaluate efficacy and safety for decision‐making, in that the study excluded patients aged over 78 years, and those with very poor glycaemic control which was defined by HbA_1c_ > 87 (10%). The study made a limited attempt to consider HTE, the only pre‐specified subgroup analysis was according to baseline HbA_1C_ and the authors did not report CATEs with confidence intervals.

We applied the additional inclusion criteria stipulated by Nauck et al. to the CPRD data to define a ‘RCT eligible’ subpopulation, which included people aged 18–78 years who had baseline HbA_1c_ of 44–87 mmol/mol (6.5%–10%) at study entry (day zero). We defined an ‘RCT ineligible’ subpopulation as the remaining subsample from the target population who did not meet the inclusion criteria for the Nauck et al. RCT.

### 4.7 Statistical analysis: Estimating ATEs and CATEs for the overall target population and for the ‘RCT eligible’ and ‘RCT ineligible’ subpopulations

We applied LIV to estimate the effects of DPP4i versus SU for each person in the entire target population. These individual effects were then aggregated to report ATE and CATEs for the overall target population, and for the ‘RCT eligible’ and ‘RCT ineligible’ subpopulations. Within the LIV estimation, the first stage models estimated the probability that each person was prescribed DDP4i given their baseline covariates and their CCG's TTP (Basu et al., 2018). The second‐stage outcome models then included the predicted probabilities from the first‐stage (propensity score) models, covariates and their interactions. Probit regression models were used to estimate the initial propensity score (first stage), while generalised linear models were applied to the outcome data, with the most appropriate family (gaussian) and link function (identity) chosen according to root mean squared error, with Hosmer‐Lemeshow and Pregibon tests also used to check model fit and appropriateness (Hosmer & Lemeshow, 2000; Pregibon, 1980). In addition to including main covariate effects in the models for both stages, we also considered the quadratic forms of both age and baseline HbA_1c_. We also considered the interaction of baseline HbA_1c_ with age, sex, and baseline BMI. We considered interactions of the IV (first‐stage models) or for the treatment indicator (second‐stage models) with baseline HbA_1c_, eGFR, BMI, SBP and age. To select the final set of interactions, we applied the rigorous Least Absolute Shrinkage and Selection Operator (LASSO) regression algorithm for the first and second stage models (Belloni et al., 2012; Frank & Friedman, 1993; Tibshirani, 2018). Following the approach detailed in (Bidulka et al., 2024), the final model specification included those variables selected by the rigorous LASSO in at least one stage.

To obtain the 95% CI of the treatment effect estimates, we bootstrapped data from the target population 500 times, stratified by geographical region and treatment group. Within each bootstrap resample, we conducted the analysis described above using the same model specification. This approach yielded 500 treatment effects (one per bootstrap resample) that were used to create confidence intervals based on a t‐distribution, as suggested by (Schomaker & Heumann, 2018).

For the *RCT‐eligible population* we compared the ATE to the corresponding estimand from the RCT with the binary agreement metric proposed by (Franklin et al., 2021) which designates *estimate agreement* if the ATE from the target trial is within the 95% CI of the RCT estimate.

#### 4.7.1 Missing data

Some measurements were missing for the HbA_1C_ outcome (31.8%) and baseline covariates (e.g., ethnicity, baseline HbA_1c_). In previous work we have showed that the estimated levels of HbA_1C_ were similar with a complete case analysis, which implies that the missingness mechanism is independent of the outcome given the covariates included in the analytical models, versus multiple imputation which assumes the data are missing at random (Bidulka et al., 2024). Hence, in this paper, we adopt the simpler approach of complete case analyses (See flow diagram in Figure S3).

#### 4.7.2 Alternative analyses

We conducted alternative analyses that avoided assuming that the IV was valid, but assumed instead that there was no unobserved confounding (selection on observables) and no essential heterogeneity. Using the same covariates as described in Sections 4.4 and 4.7, we applied inverse probability of treatment weighting with regression adjustment (IPTW‐RA; Wooldridge, 2007), which has the so‐called ‘double‐robustness property’ in that, subject to the ‘no unobserved confounding’ assumption it can still provide consistent estimates provided *either* the propensity score or the outcome regression model is correctly specified (Funk et al., 2011).

We followed the same principle as for the main analysis by using recycled predictions (Basu & Rathouz, 2005; StataCorp, 2023a) to estimate individual level effects by predicting potential outcomes (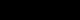) for each person with each treatment, and then calculated individual‐level differences in these predictions 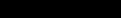, representing individualised treatment effect estimates. It is worth noting that IPTW‐RA with recycled predictions is equivalent to g‐computation regression adjustment incorporating inverse probability weighting (Smith et al., 2022). We then aggregated these individualised treatment effect estimates to each pre‐specified subgroup, and the overall populations and subpopulations of interest to obtain estimates of the CATEs and ATE.

We undertook all these alternative analyses on the same samples of complete cases as for the main analyses and the respective 95% CIs were obtained using the bootstrap approach detailed in Section 4.7. All analyses were computed using Stata 18 (StataCorp, 2023b); the results from the main analysis were estimated using the *petiv and rlasso* commands (Ahrens et al., 2020; Basu, 2015).

## 5 RESULTS

**FIGURE 2:**
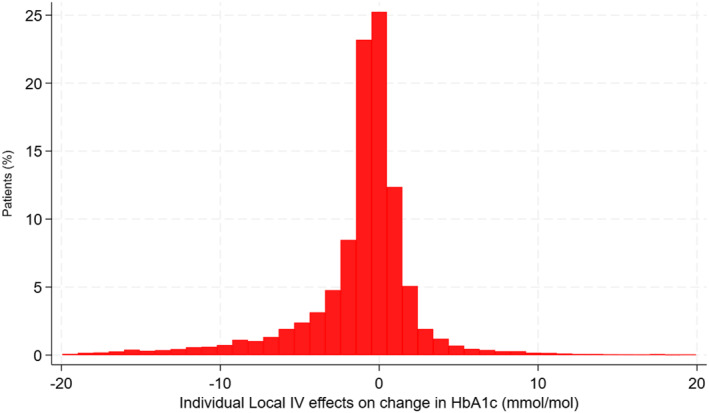
Distribution of estimated individual treatment effects reported as expected difference (DPP4i‐SUs) in change in HbA_1C_ (mmol/mol) between baseline and 1 year for the target population from the LIV approach. For presentational purposes, 135 individual effects (1% of target population) outside the range (−20, 20) mmol/mol were excluded. DPP4i, dipeptidyl peptidase‐4 inhibitors; LIV, local instrumental variable.

We identified 13,240 people from 162 CCGs in the CPRD data who met the inclusion criteria for the target population. Table 1 compares the baseline characteristics for those who had DPP4is versus SUs. For baseline measures such as age, gender and ethnicity there were only small differences between the comparison groups, but for HbA_1C_ and BMI there were important differences, those with high baseline HbA_1C_ (≥88 mmol/mol) were more likely to receive a SU, and those in obesity class 2 or 3 were more likely to have a DPP4i. Figure 2 shows the variation in the estimated individualised treatment effects across the overall target population (Figure S4 shows the distribution of these effects according to age and baseline HbA_1c_ subgroups).

**TABLE 1:** Baseline measures for the treatment groups in the target population.

Variable	Target population (*N* = 13,240)	
	SU	DPP4i
	(*n* = 8289%–62.6%)	(*n* = 4951%–37.4%)
Age: Mean (SD)	61.0 (11.7)	60.9 (11.5)
Age group years—*N* (%)		
Younger than 50	1465 (17.7%)	854 (17.3%)
50–59	2151 (26.0%)	1360 (27.5%)
60–69	2588 (31.2%)	1543 (31.2%)
70–78	1584 (19.1%)	919 (18.6%)
79 or older	501 (6.0%)	275 (5.6%)
Female *N* (%)	3290 (39.7%)	1977 (39.9%)
BMI: kg/m^2^—Mean (SD)	32.0 (6.2)	33.4 (6.4)
Under/normal weight–BMI (15–24.9)	805 (9.7%)	315 (6.4%)
Overweight–BMI (25–29.9)	2687 (32.4%)	1314 (26.5%)
Obese (class 1)–BMI (30–34.9)	2582 (31.2%)	1630 (32.9%)
Obese (class 2 & 3)–BMI (35 and higher)	2215 (26.7%)	1592 (34.2%)
Baseline HbA_1c_: mmol/mol—Mean (SD)	76.0 (19.1)	70.4 (14.8)
HbA1c distribution at baseline *N* (%)		
HbA1c < 44 mmol/mol	21 (0.3%)	11 (0.2%)
HbA_1c_ ≥ 44–63 mmol/mol	2299 (27.7%)	1891 (38.2%)
HbA_1c_ ≥ 64–74 mmol/mol	2562 (30.9%)	1648 (33.3%)
HbA_1c_ ≥ 75–87 mmol/mol	1567 (18.9%)	810 (16.4%)
HbA_1c_ ≥ 88 mmol/mol	1840 (22.2%)	591 (11.9%)
Ethnicity (%)		
White	7112 (85.8%)	4426 (89.4%)
South Asian	819 (9.9%)	371 (7.5%)
Black/mixed/other	358 (4.3%)	154 (3.1%)
Duration of diabetes: Years—Mean (SD)	5.3 (4.8)	5.8 (4.8)

Table 2 presents the LIV individualised treatment effect estimates aggregated according to the overall population (ATE) and to pre‐specified subgroups (CATE). Except for those patients in the highest baseline HbA_1C_ category, the estimated ATE and CATEs were small, and less than the thresholds for clinical or statistical significance. Table 2 reports evidence of HTE according to baseline HbA_1C_ category. For the stratum with high baseline HbA_1C_ (≥88 mmol/mol) the mean difference in HbA_1C_ in favour of DPP4i versus SU was large (−5.3) albeit with CIs that included zero (−12.8–2.2).

**TABLE 2:** ATE and CATEs for the target population from the LIV approach.

Variable	Patients per subgroup (*N*)	Treatment effect (DPP4i vs. SU)^a^ Point estimate (95% CI)
Overall	13,240	−1.3 (−3.3, 0.8)
Age group years		
Younger than 50	2319	−1.3 (−5.9, 3.3)
50–59	3511	−3.0 (−5.6, −0.4)
60–69	4131	−1.6 (−3.8, 0.6)
70–78	2503	0.6 (−1.7, 2.8)
79 or older	776	2.7 (−1.3, 6.6)
Female	5267	−1.2 (−3.1, 0.8)
BMI (kg/m^2^)		
Under/normal weight–BMI (15–24.9)	1120	−0.2 (−3.6, 3.1)
Overweight–BMI (25–29.9)	4001	−0.5 (−2.9, 1.9)
Obese (class 1) –BMI (30–34.9)	4212	−1.1 (−3.1, 0.9)
Obese (class 2 & 3)–BMI (35 and higher)	3907	−2.5 (−5.2, 0.1)
HbA1c distribution at baseline		
HbA1c < 44 mmol/mol	32	−2.2 (−10.4, 6.0)
HbA_1c_ ≥ 44–63 mmol/mol	4190	−0.1 (−1.8, 1.6)
HbA_1c_ ≥ 64–74 mmol/mol	4210	−0.3 (−2.0, 1.5)
HbA_1c_ ≥ 75–87 mmol/mol	2377	−1.1 (−3.9, 1.7)
HbA_1c_ ≥ 88 mmol/mol	2431	−5.3 (−12.8, 2.2)
Ethnicity		
White	11,538	−1.3 (−3.3, 0.7)
South Asian	1190	−0.7 (−3.5, 2.1)
Black/‐mixed/other	512	−1.5 (−5.1, 2.0)

^a^ Difference in the change in HbA_1c_ (mmol/mol) from baseline.

Table 3 presents the baseline characteristics from the Nauck et al. RCT versus the subpopulations from the CPRD data who met the RCT eligibility criteria (RCT‐eligible), and for those did not meet these criteria (RCT‐ineligible) as well as for the overall target populations. By definition, the ‘RCT‐eligible population’ excluded the stratum with the older age group and higher baseline HbA_1c_. The RCT‐eligible sample were representative of the broader target population according to characteristics not used to define eligibility criteria such as BMI, gender and ethnicity. However, mean baseline HbA_1C_ was much lower in the Nauck et al. RCT and the corresponding ‘RCT‐eligible’ subpopulation, than for the ‘RCT‐ineligible’ population.

**TABLE 3:** Baseline measures by treatment group for the Benchmark RCT (shaded), and for the ‘RCT eligible’, ‘RCT ineligible and overall target populations from the CPRD data (target trials).

Variable	Benchmark RCT Nauck et al. (N = 1172)		RCT‐eligible population (N = 6497)		Target trial RCT ineligible population (N = 6743)		Target population (N = 13,240)	
	SU	DPP4i	SU	DPP4i	SU	DPP4i	SU	DPP4i
	(n = 588–50.2%)	(n = 584–49.8%)	(n = 3931%–60.5%)	(n = 2566%–39.5%)	(n = 4358%–64.6%)	(n = 2385%–35.4%)	(n = 8289%–62.6%)	(n = 4951%–37.4%)
Age: Years—Mean (SD)	56.6 (9.8)	56.8 (9.3)	60.8 (10.4)	60.3 (10.2)	61.2 (12.8)	61.6 (12.7)	61.0 (11.7)	60.9 (11.5)
Age subgroups N (%)								
Younger than 50			614 (15.6%)	426 (16.6%)	851 (19.5%)	428 (18.0%)	1465 (17.7%)	854 (17.3%)
50–59	*	*	1035 (26.3%)	726 (28.3%)	1116 (25.6%)	634 (26.6%)	2151 (26.0%)	1360 (27.5%)
60–69	*	*	1385 (35.2%)	883 (34.4%)	1203 (27.6%)	660 (27.7%)	2588 (31.2%)	1543 (31.2%)
70–78	*	*	897 (22.8%)	531 (20.7%)	687 (15.8%)	388 (16.3%)	1584 (19.1%)	919 (18.6%)
79 or older	Ineligible	Ineligible	Ineligible	Ineligible	501 (11.5%)	275 (11.5%)	501 (6.0%)	275 (5.6%)
Female (%)	226 (38.7%)	252 (42.9%)	1579 (40.2%)	1012 (39.4%)	1711 (39.3%)	965 (40.5%)	3290 (39.7%)	1977 (39.9%)
BMI: kg/m^2^—Mean (SD)	31.3 (5.2)	31.2 (5.0)	32.2 (6.1)	33.6 (6.3)	31.8 (6.3)	33.1 (6.5)	32.0 (6.2)	33.4 (6.4)
BMI subgroups N (%)								
Under/normal weight	*	*	337 (8.6%)	132 (5.1%)	468 (10.7%)	183 (7.7%)	805 (9.7%)	315 (6.4%)
Overweight	*	*	1289 (32.8%)	669 (26.1%)	1398 (32.1%)	645 (27.04%)	2687 (32.4%)	1314 (26.5%)
Obese (class 1)	*	*	1237 (31.5%)	867 (33.8%)	1345 (30.9%)	763 (32.0%)	2582 (31.2%)	1630 (32.9%)
Obese (class 2 & 3)	*	*	1068 (27.2%)	898 (35.0%)	1147 (26.3%)	794 (33.3%)	2215 (26.7%)	1592 (34.2%)
Baseline HbA_1c_: mmol/mol—Mean (SD)	58.8 (6.6)	58.4 (5.9)	68.8 (8.3)	67.4 (8.2)	82.5 (23.3)	73.6 (19.1)	76.0 (19.1)	70.4 (14.8)
Baseline HbA1c subgroups N (%)								
HbA1c < 44 mmol/mol	Ineligible	Ineligible	Ineligible	Ineligible	21 (0.5%)	11 (0.5%)	21 (0.3%)	11 (0.2%)
HbA_1c_ ≥ 44–63 mmol/mol	381 (65.5%)	375 (64.0%)	1225 (31.1%)	993 (38.7%)	1074 (24.6%)	898 (37.7%)	2299 (27.7%)	1891 (38.2%)
HbA_1c_ ≥ 64–74 mmol/mol	141 (24.2%)	151 (25.8%)	1674 (42.6%)	1053 (41.0%)	888 (20.4%)	595 (25.0%)	2562 (30.9%)	1648 (33.3%)
HbA_1c_ ≥ 75–87 mmol/mol	60 (10.3%)	60 (10.2%)	1032 (26.3%)	520 (20.3%)	535 (12.3%)	290 (12.2%)	1567 (18.9%)	810 (16.4%)
HbA_1c_ ≥ 88 mmol/mol	Ineligible	Ineligible	Ineligible	Ineligible	1840 (42.2%)	591 (24.8%)	1840 (22.2%)	591 (11.9%)
Ethnicity N (%)								
White	74.3%	73.5%	3421 (87.0%)	2315 (90.2%)	3691 (84.7%)	2111 (88.5%)	7112 (85.8%)	4426 (89.4%)
South Asian	8.4%	8.5%	379 (9.6%)	185 (7.2%)	440 (10.1%)	186 (7.8%)	819 (9.9%)	371 (7.5%)
Black/mixed/other	17.3%	18.0%	131 (3.3%)	66 (2.6%)	227 (5.2%)	88 (3.7%)	358 (4.3%)	154 (3.1%)
Duration of diabetes: Years—Mean (SD)	6.5 (6.1)	6.2 (5.4)	5.5 (4.7)	5.9 (5.0)	5.2 (4.9)	5.6 (4.7)	5.3 (4.8)	5.8 (4.8)

For the target trials there were some differences between the treatment groups in observed baseline characteristics. For the ‘RCT‐eligible’ subpopulation, most strata were balanced, but there were between‐treatment group differences in the proportion of patients in obesity class 2 and 3, and in baseline HbA_1c_ ≥75–87. As anticipated, baseline imbalances were more pronounced for the ‘RCT‐ineligible’ subpopulation, with large between‐treatment differences in the proportions with HbA1C ≥ 88 and in the proportions in Obesity Classes 2 and 3. These observed measures may be correlated with unobserved characteristics such as dietary patterns, levels of exercise and adherence to previous medications, which increases the risk of unobserved confounding and also essential heterogeneity in the ‘RCT ineligible’ subpopulation.

**FIGURE 3:**
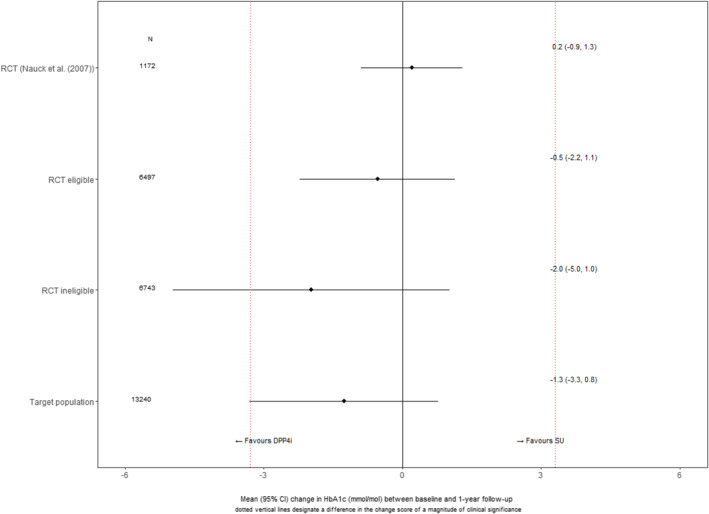
ATEs from Nauck et al. (2007), and for the corresponding ‘RCT eligible’ subpopulation, ‘RCT eligible’ and overall target populations from the target trial using the LIV approach. Average Treatment effects (ATEs) reported as difference (DPP4i‐SUs) in change in HbA_1C_ (mmol/mol) between baseline and 1 year. DPP4i, dipeptidyl peptidase‐4 inhibitors; RCT, randomised controlled trials; LIV, local instrumental variable.

**FIGURE 4:**
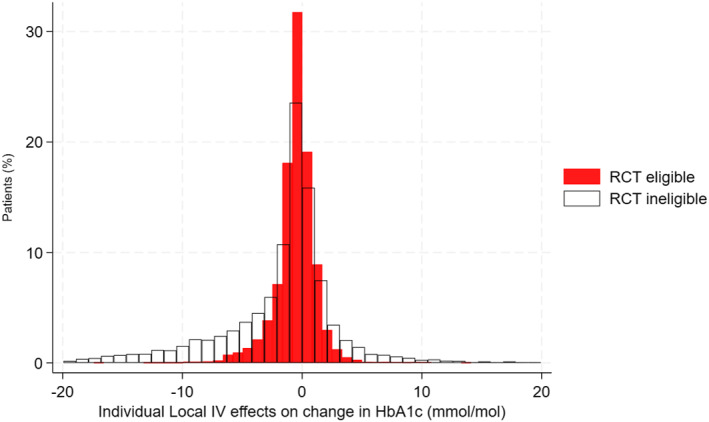
Distribution of estimated individual treatment effects reported as expected difference (DPP4i‐SUs) in change in HbA_1C_ (mmol/mol) between baseline and 1 year, for the ‘RCT eligible’ versus ‘RCT ineligible’ subpopulations. For presentational purposes, 135 individual effects (all from the RCT ineligible population and accounting for 1% of target population) outside the range (−20, 20) mmol/mol were excluded. DPP4i, dipeptidyl peptidase‐4 inhibitors; RCT, randomised controlled trials.

Figure 3 compares the ATE from the Nauck et al. RCT to the appropriate ATE from the LIV method, which comprise of individual‐level treatment effects aggregated to the ‘RCT‐eligible’ subpopulation. Neither of the estimated ATEs were of ‘clinical’ or ‘statistical’ significance. The point estimates for the ‘RCT‐eligible’ population were within the 95% CIs of those for the RCT, indicating ‘estimate agreement’. As the RCT eligibility criteria meant that there were important differences in baseline characteristics that were anticipated to modify the treatment effect, the ATEs for the ‘RCT‐ineligible’ and the ‘target’ populations were not directly comparable to those for the Nauck et al. RCT, but are reported here for completeness. The ATE estimate for the ‘RCT‐ineligible’ population was more uncertain than for the ‘RCT‐eligible’ population which reflected greater variation in the individualised treatment effect estimates across the broader patient group, which included those who failed the eligibility criteria according to observed levels of baseline HbA_1C_ and age (see Figure 4 and corresponding figures for these two baseline measures in the supplement).

The alternative analysis assuming no unobserved confounding provided estimates of ATEs that were in the opposite direction to those from the LIV, but for the ‘RCT‐eligible’ population still met the criteria for ‘estimate agreement’ with those from Nauck et al. The alternative analyses like the LIV reported more uncertainty in the estimates for the ‘RCT‐ineligible’ versus ‘RCT‐eligible’ subpopulations. While the estimated ATEs from IPTW‐RA are statistically different from zero, none of them were of a magnitude that met the criteria for clinical significance (See Figure S5 in the Supplement).

**FIGURE 5:**
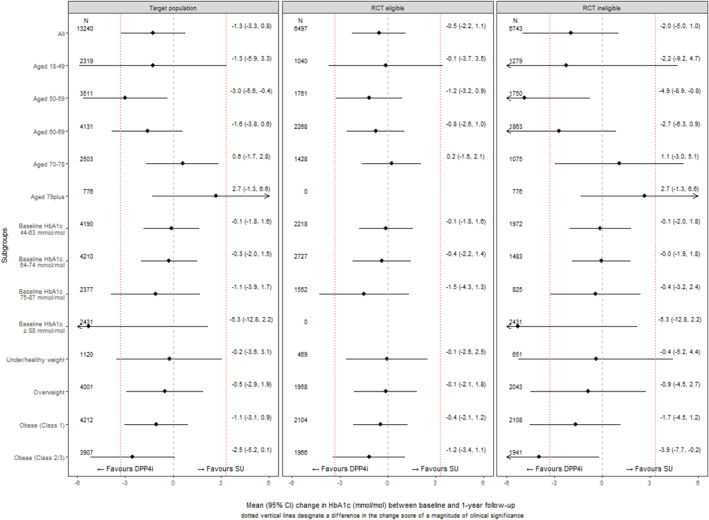
Conditional Average Treatment Effects (CATEs) for the ‘RCT eligible’ subpopulation, ‘RCT eligible’ and overall target populations from the target trial using the LIV approach for age and baseline HbA_1c_ subgroups. CATEs reported as difference (DPP4i‐SUs) in change in HbA_1C_ (mmol/mol) between baseline and 1 year. DPP4i, dipeptidyl peptidase‐4 inhibitors; RCT, randomised controlled trials; LIV, local instrumental variable.

Figure 5 reports the CATE estimates from the LIV method for the ‘RCT‐eligible’ and ‘RCT‐ineligible’ subpopulations as well as for the target population. The results show some evidence of HTE in particular according to baseline HbA_1C_, age group, and BMI (additional subgroups available in Supplementary Figures S6–S8). The estimated CATEs were in similar directions for the ‘RCT‐eligible’ and ‘RCT‐ineligible’ subpopulations and therefore for the target populations. For some subgroups, the magnitude of the estimated CATEs within the ‘RCT‐ineligible’ subpopulation were somewhat different to those for the ‘RCT‐eligible’ population, and the estimated CATEs differed between the subgroups excluded from the RCT versus those included. In particular, for people with high levels of baseline HbA1C (≥88 mmol/mol) the estimated improvement in HbA_1C_ following DPP4i versus SU was of clinical significance, albeit estimated with high levels of uncertainty.

## 6 DISCUSSION

Target populations for decision‐making may differ from those eligible for RCTs according to baseline characteristics that may modify the relative effectiveness of health care interventions. We use a target trial emulation with an LIV method to enable us to fully examine treatment effect heterogeneity including effect modification according to levels of unobserved covariates (essential heterogeneity) across subpopulations eligible and ineligible for a published RCT. We consider the approach within a case study evaluating the effectiveness of two alternative second‐line treatments for people with T2DM. We applied the LIV method to estimate individualised treatment effects, that we then aggregated to report ATEs and CATEs across the full target population, defined by a national clinical guideline, and for the ‘RCT‐eligible’ and ‘RCT‐ineligible’ subpopulations. The estimated ATEs for the ‘RCT‐eligible’ population are similar to those from a published RCT. The estimated CATEs are in the same direction for the subpopulations included versus excluded from the RCT, but differ in magnitude. The variation in the estimated individual treatment effects is greater across the broader sample of people who do not meet the RCT inclusion criteria than for those who do.

This paper contributes to three related areas of research: the transportability of results from RCT eligible populations to target populations for decisions, essential heterogeneity, and emulating target trials. First, previous work has developed methods for transporting estimates of ATEs and CATEs from RCTs to a target population, with recent expansions including the use of flexible machine learning methods (Allcott & Mullainathan, 2012; Dahabreh & Hernán, 2019; Degtiar & Rose, 2023; Elliott et al., 2023; Hartman et al., 2015; Stuart et al., 2010). This extant literature has made the common crucial assumption, that there is no essential heterogeneity, which can also be expressed as no trial selection according to unobserved variables that modify the relative treatment effect. The plausibility of this assumption will depend on the setting, and will relate to issues around the RCT design and the availability of common baseline measures between the RCT and target population. An RCT with a more pragmatic design may impose less restrictive eligibility criteria and be less prone to select participants according to unobserved characteristics that are likely to modify the treatment effect. Also, an RCT nested within a data source such as a disease registry that collects a common set of baseline variables including all potential effect modifies is better placed to transport the RCT result. More generally, given the non‐random selection of RCT participants, those included in RCTs are likely to differ according to measures that are not fully observed in the RCT(s) or observational data, including those correlated with the explicit inclusion criteria. For example, in our case study, people with poor baseline glycaemic control were excluded which is correlated with diet and previous adherence to medication, neither of which were observed in the EHR or RCT data (Nauck et al., 2007; Zaccardi et al., 2020). Similarly, older patients were excluded which is correlated with frailty, which was also not observed. Hence, an RCT finding, in this case of no significant differences in clinical effectiveness between the alternative treatments, may not transport to the target population and subpopulations. The approach taken provides new evidence, for policy relevant subgroups excluded from the RCT.

Second, the paper contributes to the literature on assessing essential heterogeneity. Previous work has developed conceptual frameworks (Cornelissen et al., 2016; Heckman and Vytlacil, 1999, 2001, 2005) for understanding essential heterogeneity in general settings, shown how under standard IV assumptions and with a continuous IV the requisite marginal treatment effects can be estimated (Heckman and Vytlacil, 1999, 2001), and how these can be aggregated to estimate policy‐relevant estimands including the ATE and CATE (Basu, 2014, 2015; Heckman & Vytlacil, 2001). The LIV approach taken in this paper has been applied across a diverse range of settings, including to assess the effects of interventions in education as well as health care (Basu, 2014; Basu et al., 2007, 2014; Basu & Gore, 2015; Grieve et al., 2019). A recent simulation study showed that in the presence of both overt and essential heterogeneity, the LIV approach taken in this paper can report consistent effect estimates of ATEs and CATEs provided the IV is sufficiently strong (F statistic>100), and the sample size sufficiently large (>5000) (Moler‐Zapata et al., 2023a). This paper adds to this literature by using target trial emulation to assess the performance of the approach when an ATE from a RCT is available for a subpopulation, and to explore essential heterogeneity across an entire target population including subpopulations who met RCT eligibility criteria, and those who did not. The approach taken has wider application to settings where the likely presence of essential heterogeneity raises challenges for the transportability of RCT(s) results to the target populations of interest.

Third, the paper contributes to the literature emulating target trials for decision‐making. Previous studies have applied aspects of the approach we take, in using a target trial design, firstly to emulate a published RCT, and secondly to estimate ATEs for a population excluded from the RCT. However, these previous target trials have only applied analytical methods that assume ‘no unobserved confounding’. In our study we found that for the ‘RCT‐eligible subpopulation’ a method that assumes ‘no observed confounding’ (IPTW‐RA) provides similar ATE estimates to those from the LIV approach and to the RCT benchmark, and also similar CATE estimates to the LIV approach. By contrast for the RCT‐ineligible population, there were wide baseline imbalances according to observed potential confounders, such as age, which were likely correlated with unobserved confounders such as frailty. Hence, this is a more challenging setting for approaches that assume no unobserved confounding and no essential heterogeneity as it is likely these assumptions are implausible, and this may explain why the estimated CATEs from the IPTW‐RA approach differed to those from the LIV method. Moreover, there was less variation in the estimated individualised treatment effects when they were calculated using the IPTW‐RA approach versus the LIV. Hence, while the LIV estimates of the CATEs and ATEs are less precise than those for the IPTW‐RA this may partly reflect the appropriate capture of essential heterogeneity. Future target trial approaches should consider LIV approaches that can address both confounding and heterogeneity, and avoid relying on methods that assume no unobserved confounding and no essential heterogeneity, especially when a valid instrument is available and interest lies in broader populations for which there is no RCT benchmark.

This paper is subject to some limitations. First, we explored HTE within a single clinical scenario which cannot cover all the features that arise in practice when attempting to transport comparative effectiveness evidence from RCT eligible populations to target populations. In other settings, where an RCT requiring informed consent is nested within the target population, this may imply somewhat different selection mechanisms, and imply further challenges when transporting the trial results to the target population. While the potential importance of deploying approaches that consider essential heterogeneity remain, an extra problem is that the form of treatment may differ between the specific protocols required for the RCT versus those used in practice. Second, the comparators of interest in the target population may not be included in any particular RCT. In response to this concern a network meta‐analyses may include RCTs with the relevant comparators. Here, the challenge of transporting results from the RCT setting to the target population is somewhat different as the RCTs may well have different eligibility criteria, and so the approach taken here would need to be extended to consider HTE across the target population including subpopulations who do meet and do not meet the eligibility criteria for different RCTs within the network. Third, this paper considered a single endpoint, but the approach could be applied to multiple endpoints recognising that HTE may differ across the different outcomes. Fourth, in our example, we had a strong IV and moderately large sample size, but the LIV estimates that incorporated essential heterogeneity were still somewhat imprecise. Stein‐like approaches that combine efficient but inconsistent estimators (e.g., ordinary least squares) with consistent but inefficient estimators (e.g., 2SLS/LIV) to improve precision at the expense of a somewhat higher risk of bias may warrant consideration for estimation of HTE (Hansen, 2017). This may be particularly useful for studies focussing on smaller subgroups or that have weaker instruments.

Future research is required to explore HTE in populations explicitly excluded from RCTs, across a broader array of settings including those where the RCT is nested within the EHR data to allow this aspect of selection to be formally studied. It would also be helpful to consider settings with multiple outcomes, only some of which may be available in the RCT with others in the EHR data, and in settings where the issue is in transporting findings from network meta‐analyses of RCTs to a target population. In settings, where more than two treatment comparators are of interest, further development of the requisite LIV methods are required as current approaches identify the marginal treatment effect of a treatment versus the next best alternative (Heckman et al., 2008), but do not readily provide CATE estimates for specific treatment comparisons.

## CONFLICT OF INTEREST STATEMENT

PB, OC, SON, AIA, and KDO have no disclosures. DLP served as external consultant for Blutitude Consultores on a project not related to diabetes. AB reports Personal Fees from Salutis Consulting LLC outside the scope of the submitted work, AHB reports grants from NIHR during the conduct of the study and personal fees outside the submitted work from Boehringer Ingelheim, Novartis, Takeda, Idorsia, Rhythm, Daiichi‐Sankyo, Bayer, GSK, and Gilead. RG reports grants from NIHR during the conduct of the study.

## Supporting information

Supporting Information S1

## Data Availability

The data that support the findings of this study are available from UK Medicines and Healthcare products Regulatory Agency (MHRA. Restrictions apply to the availability of these data, which were used under licence for this study. Data are available from https://www.cprd.com/ with the permission of UK Medicines and Healthcare products Regulatory Agency (MHRA).
