# Supplementary material for: Going beyond randomised controlled trials to assess treatment effect heterogeneity across target populations
Source: Health Econ. 2024 Sep 26;34(1):85–104. doi: 10.1002/hec.4903 (PMC11631826; doi:10.1002/hec.4903)
Supplement: Supplementary file 1 — Supporting Information S1 [file HEC-34-85-s001.docx]

**Supplementary Material**

**Going beyond Randomised Controlled Trials to assess treatment effect heterogeneity across target populations**

**Table S1.** Protocol to emulate the Nauck et al. (2007) RCT using CPRD data.


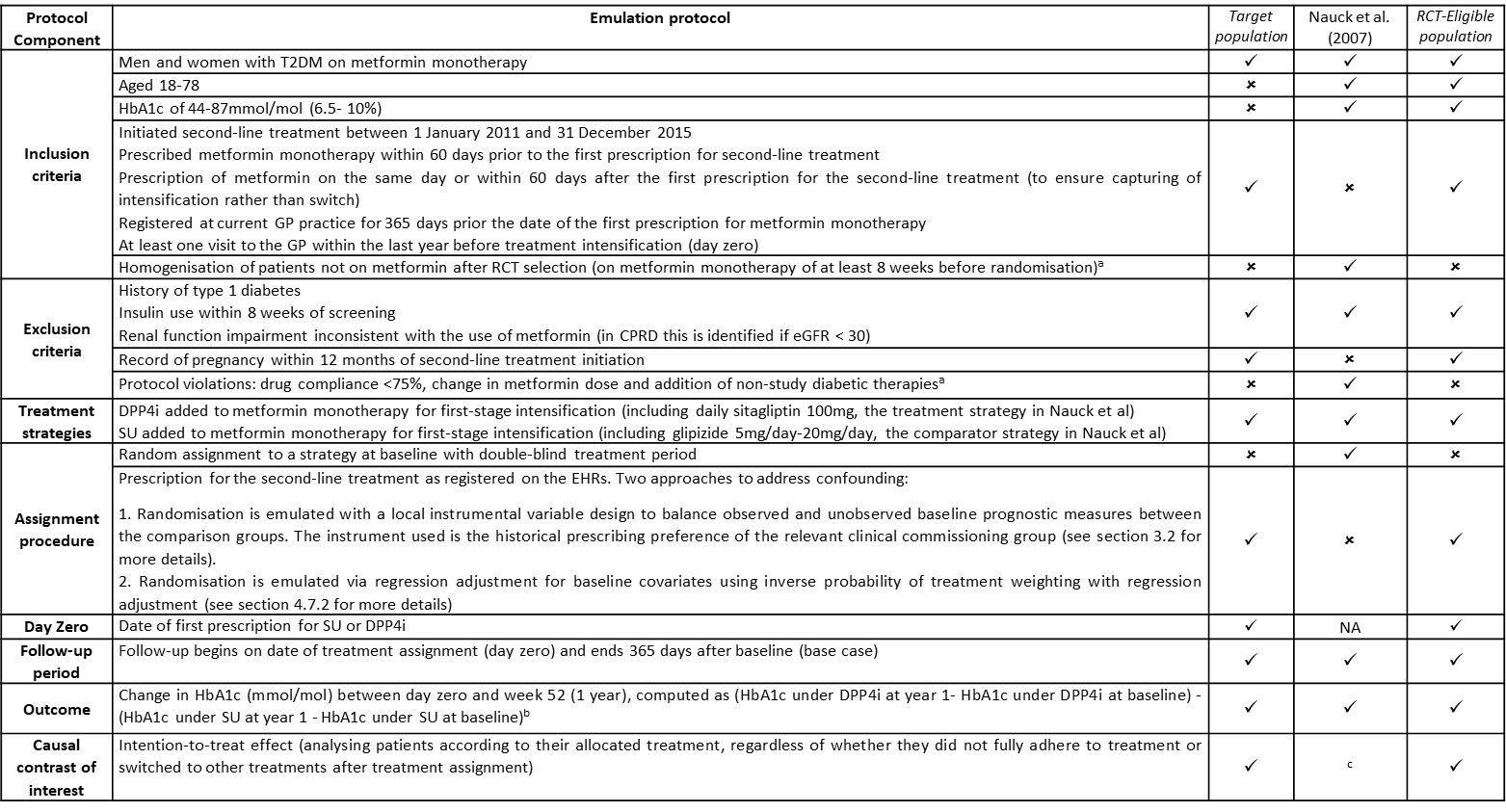


^a^ Not possible to emulate using CPRD data. ^b^ Negative values indicate higher effect of DPP4is on HbA1c at 1 year and positive values indicate higher effect of SUs. Nauck et al measures HbA1c in %, but results are transformed to mmol/mol: (HbA1c (%) - 2.14) x 10.929 [33]. ^c^ The Nauck et al. (2007) per protocol (PP) population was used as a contrast as more details were reported for this population; the results reported in Nauck et al. for the ‘all-patients-treated’ population supported those for the PP population.

**Table S2:** Details of covariate data sources and definitions

| **Covariable** | **Data source** | **Details** |
| --- | --- | --- |
| Age | CPRD | CPRD  Age at baseline derived using the year of birth |
| Sex | CPRD | CPRD  Sex recorded in CPRD |
| Ethnicity | CPRD, HES | CPRD  Clinical code (Read or Snomed) indicating ethnicity, further categorised into four categories (White, South Asian, Black, Mixed/Other)  HES  Demographic data entered at in-patient hospitalisation, further categorised into four categories (White, South Asian, Black, Mixed/Other)  Where CPRD ethnicity is missing, HES ethnicity is used to define people’s ethnicity. Where ethnicities disagree, that recorded in CPRD is used. |
| Time since type 2 diabetes diagnosis | CPRD | CPRD  Days between the first diagnosis code (Read or Snomed) for T2DM and baseline |
| Time on first-line (metformin monotherapy) | CPRD | CPRD  Days between the first prescription for metformin and baseline |
| GP size | CPRD | CPRD  Number of patients actively registered with the GP to which the patient belongs, derived using the CPRD denominator file, uses 2014 figures |
| NHS Region | CPRD | CPRD  The region in which the GP practice is located to which each patient is registered. Regions include: East of England, London, Midlands, North East and Yorkshire, North West, South East, and South West |
| Co-prescriptions prescribed within 60 days of baseline (including RASi and statins) | CPRD | CPRD  At least one prescription for the drug class of interest in the prescription history in the primary care record, within 60 days of baseline. |
| Comorbidities at baseline defined in primary and secondary care (including previous MI, unstable angina, stroke, hypoglycaemia, CHF) | CPRD and HES | CPRD  Diagnosis code (Read or Snomed) for each comorbidity prior to or the same day as baseline  HES  Diagnosis code (ICD-10) for each comorbidity prior to or the same day as baseline in any diagnostic position of any episode of a spell |
| Comorbidities at baseline defined in primary care (cancer (any), advanced eye disease, lower extremity amputation, proteinuria) | CPRD | CPRD  Diagnosis code (Read or Snomed) for comorbidity prior to or the same day as baseline |
| HbA1c | CPRD | CPRD  Laboratory test recording the most recent HbA1c recorded within 180 days prior to baseline. Units reported as mmol/mol (tests recording HbA1c in % will be converted to mmol/mol). |
| eGFR and eGFR/CKD status | CPRD | CPRD  Using the eGFR derived from serum creatinine using the CKD-EPI equation without adjustment for ethnicity recorded within 540 days prior to baseline, we will group people as either having eGFR≥60mL/min/1.73m^2^ or eGFR<60mL/min/1.73m^2^ (indicating impaired kidney function) |
| SBP and DBP | CPRD | CPRD  Clinical measures captured in CPRD within 540 days prior to baseline |
| BMI | CPRD | CPRD  BMI derived from weight and height measures entered by the GP (preferred), or BMI entered directly by the GP |
| Smoking status | CPRD | CPRD  Clinical codes describing smoking status in the primary care record, using an algorithm previously defined in CPRD data |
| Alcohol status | CPRD | CPRD  Clinical codes describing alcohol intake in the primary care record, using an algorithm previously defined in CPRD data |
| In-patient hospitalisation (any reason) in the past year | HES | HES  At least one spell (hospitalisation) recorded in the patient’s secondary care record (HES admitted patient care record) in the year prior to baseline |

**Note S1:** Description of the trial review screening process to identify suitable trials for this target trial emulation

We used a list of published trials which directly compared DPP4i vs SU among people with type 2 diabetes mellitus (T2DM) (**Hanlon et al., 2024, see** https://doi.org/10.1101/2024.06.23.24309242). This list included 35 published trials. We screened for trials eligible for this target trial emulation in the following two-step process (see also **Table S3a**):

1. Screening the title, abstract, and methods sections to ensure the trials were phase 3, double-blind trials among a general population of people with T2DM and reported HbA1c at baseline and at 52 weeks follow-up (1-year) as an outcome. Those which did not meet these criteria were excluded at this stage.
2. Among those passing the first screening, the methods were reviewed in further detail to ensure the trial met the following criteria:
   1. Results were reported as intention-to-treat in the primary analysis.
   2. Missing outcome data (HbA1c at 1-year) were accounted for with rigorous methodology (e.g., not last observation carried forward).
   3. Did not exclude participants based on criteria we determined in advance were difficult to define in the Clinical Practice Research Datalink (CPRD) (e.g., liver laboratory test results, family history of disease, clinical judgements).
   4. Did not exclude a significant proportion of people based on a clinical characteristic so as to make the study population not representative of a general T2DM population (e.g., exclude those with a history of cancer).
   5. Outcome data were reported in tables with exact numbers (i.e., HbA1c at baseline and 1-year were not only reported in figure format).
   6. Peer-reviewed publication was accessible online.

Of the 35 published trials which directly compared DPP4i vs SU, 9 trials passed the first screening and only 1 trial passed the second screening (**Table S3b**). Reasons for not passing the first and second screening are presented in **Table S3b**.

Briefly, of the 26 trials which did not pass the first screening, 18 did not report HbA1c at 1-year follow-up, 8 were not in a general T2DM population, 3 were not double-blinded, 10 were not phase 3 trials, and 1 compared adding on DPP4i to SU vs only SU. Reasons for exclusion were not mutually exclusive.

Briefly, of the 8 trials which did not pass the second screening, 1 was a per protocol analysis, 1 used last HbA1c measure carried forward to impute missing HbA1c outcome data, 1 only presented HbA1c outcome data in a figure (no exact numbers reported in tables), 4 applied exclusion criteria which reduced the representativeness of the study population or were difficult to define in CPRD data (history of cancer, family history of medullary thyroid carcinoma, liver function tests, clinical judgement on risk of dehydration of volume depletion), and 1 trial did not have an accessible publication.

One trial by Nauck et al passed the two screens and was therefore used in this target trial emulation.

**Table S3a:** Description of the trial review screening process to identify suitable trials for this target trial emulation

| **Screening step** | **Details** |
| --- | --- |
| Screen 1 | Review trial registration (e.g., ClinicalTrials.gov) and exclude trials which:   - Are not phase 3 trials. - Are not double-blind (e.g., open-label). - Are not in the general type 2 diabetes mellitus (T2DM) population (e.g., include only elderly patients, patients with chronic kidney disease). - Do not report HbA1c at 52-weeks (1-year) as an outcome. |
| Screen 2 | Review trial registration and published peer-reviewed papers and exclude trials which:   - Analyse results with a per-protocol analysis as the primary analysis. - Impute missing outcome data (HbA1c at 1-year) without rigorous methodology (e.g., last observation carried forward). - Exclude based on criteria difficult to define in the Clinical Practice Research Datalink (CPRD) (e.g., liver laboratory test results, family history of disease, clinical judgements). - Exclude people with a history of cancer. - Only report changes in HbA1c at 1-year using a figure (i.e., cannot extract precise outcome measures). - Peer-reviewed article not available online. |

**Table S3b:** Summary of randomised controlled trial (RCT) search for target trial emulation

| **Trial ID** | **Trial phase** | **Study population** | **Change in HbA1c at 1 year reported** | **Screen 1** | **Exclusion justification** | **Screen 2** | **Exclusion justification** |
| --- | --- | --- | --- | --- | --- | --- | --- |
| NCT00094770 | 3 | T2DM | 1 | 1 | - | 1 | - |
| NCT00575588 | 3 | T2DM | 1 | 1 | - | 0 | Per protocol analysis. |
| NCT00622284 | 3 | T2DM | 1 | 1 | - | 0 | Used last measure carried forward to impute missing outcome values in primary analysis. |
| NCT00838903 | 3 | T2DM | 1 | 1 | - | 0 | Exclusion criteria include family history of medullary thyroid carcinoma or multiple endocrine neoplasia type 2 and liver laboratory test results difficult to define in CPRD. |
| NCT00856284 | 3 | T2DM | 1 | 1 | - | 0 | Exclusion criteria include history of cancer. |
| NCT01682759 | 3 | T2DM | 1 | 1 | - | 0 | Cannot access article. |
| NCT02471404 | 4 | T2DM | 1 | 1 | - | 0 | Exclusion criteria include clinical judgement for risk of dehydration or volume depletion. |
| NCT00102466 | 3 | T2DM | 1 | 1 | - | 0 | Liver laboratory test results difficult to define in CPRD. |
| NCT01794143 | 3 | T2DM | 0 | 1 | - | 0 | Precise HbA1c at 52 weeks not available (figure only). |
| NCT00102388 | 3 | T2DM | 0 | 0 | No HbA1c at 1 year. | - | - |
| NCT00707993 | 3 | T2DM and elderly (65-90 years) | 1 | 0 | Not general T2DM population. | - | - |
| NCT01006603 | phase 3b/4 | T2DM and elderly (65+ years) | 1 | 0 | Not general T2DM population. | - | - |
| NCT01204294 | 3 (open-label) | T2DM | 1 | 0 | Open-label trial. | - | - |
| NCT01243424 | 3 | T2DM and CVD | 0 | 0 | Not general T2DM population, and no HbA1c at 1 year (48 or 64 weeks only reported as a figure). | - | - |
| NCT00509236 | 3 | T2DM and ESKD | 1 | 0 | Not general T2DM population. | - | - |
| NCT00509262 | 3 | T2DM and CKD | 1 | 0 | Not general T2DM population. | - | - |
| NCT00701090 | 3 | T2DM | 0 | 0 | No HbA1c at 1 year. | - | - |
| NCT01183104 | 4 (open-label) | T2DM and elderly (60+ years) and not on any other antidiaebtic, incl metformin | 1 | 0 | Not general T2DM population and open-label. | - | - |
| NCT01189890 | 3 | T2DM and elderly (65-85 years) | 0 | 0 | Not general T2DM population. | - | - |
| NCT01822548 | 3 |  | 0 | 0 | No HbA1c at 1 year. | - | - |
| NCT01871558 | 3 |  | 0 | 0 | No HbA1c at 1 year. | - | - |
| NCT02007278 | 4 |  | 0 | 0 | No HbA1c at 1 year and phase 4. | - | - |
| NCT00106340 | 3 | T2DM | 0 | 0 | No HbA1c at 1 year. | - | - |
| NCT00957060 | 4 | T2DM | 0 | 0 | No HbA1c at 1 year and phase 4. | - | - |
| NCT01099137 | 4 | T2DM | 0 | 0 | No HbA1c at 1 year and phase 4. | - | - |
| NCT01341717 | 4 | T2DM | 0 | 0 | No HbA1c at 1 year and phase 4. | - | - |
| NCT01547104 | 4 | T2DM | 0 | 0 | No HbA1c at 1 year and phase 4. | - | - |
| NCT01847144 | 4 | T2DM | 0 | 0 | No HbA1c at 1 year and phase 4. | - | - |
| NCT02280486 | 4 (open-label) | T2DM | 0 | 0 | No HbA1c at 1 year and phase 4 open-label. | - | - |
| NCT03693560 | 4 | T2DM | 0 | 0 | No HbA1c at 1 year and phase 4. | - | - |
| Eudra CT 2004-004559-21 | 3 | T2DM | 0 | 0 | No HbA1c at 1 year. | - | - |
| UMIN000004791 | 3 | T2DM | 1 | 0 | Adding on DPP4i to SU, not comparing. | - | - |
| UMIN000006986 | 4 | T2DM | 0 | 0 | No HbA1c at 1 year and phase 4. | - | - |
| UMIN000009544 | 4 (open-label) | T2DM | 0 | 0 | No HbA1c at 1 year and phase 4 open-label. | - | - |
| UMIN000013356 | 4 (open-label) | T2DM with BMI >=25kg/m2 or fatty liver | 0 | 0 | No HbA1c at 1 year and phase 4 (open-lavel) and not general T2DM population. | - | - |

**Figure S1.** Directed acyclic graph (DAG) illustrating the causal relationship between the instrument, exposure, and HbA1c from baseline to 1-year follow-up

The directed acyclic graph below illustrates that the receipt of second-line treatment is subject to unmeasured and (context- and individual-level) observed factors that confounds the link between treatment and the outcome of interest (biomarkers at 1 year). This figure suggests that the clinical commissioning group’s (CCG’s) tendency to prescribe (the proposed instrumental variable) predicts the second-line treatment received by a patient registered in that CCG, but does not have a direct effect on the health outcome of interest. That is, it is assumed that the only path through which the CCGs tendency to prescribe influences the biomarkers at 1 year is through its influence in the treatment received. Thus, this reflects the explicit assumption of an IV design that the instrument is not independently associated with outcomes, unobserved confounders and individual-level confounders. The DAG allows for an association between context-level confounders (such as GP practice list size) and the IV as larger practices may have different prescription patterns compared to smaller practices. The individual-level confounders considered in this hypothesised causal diagram were classified in three broad categories: patient’s socio-demographic characteristics (age, sex, etc.), baseline health status (e.g. relevant comorbidities, biomarkers and medications such as statins and renins), and baseline behaviour (alcohol and smoking status). For simplicity, this figure does not reflect all the existing correlations amongst different factors, for example the one existing between unobserved and individual-level confounders.

**
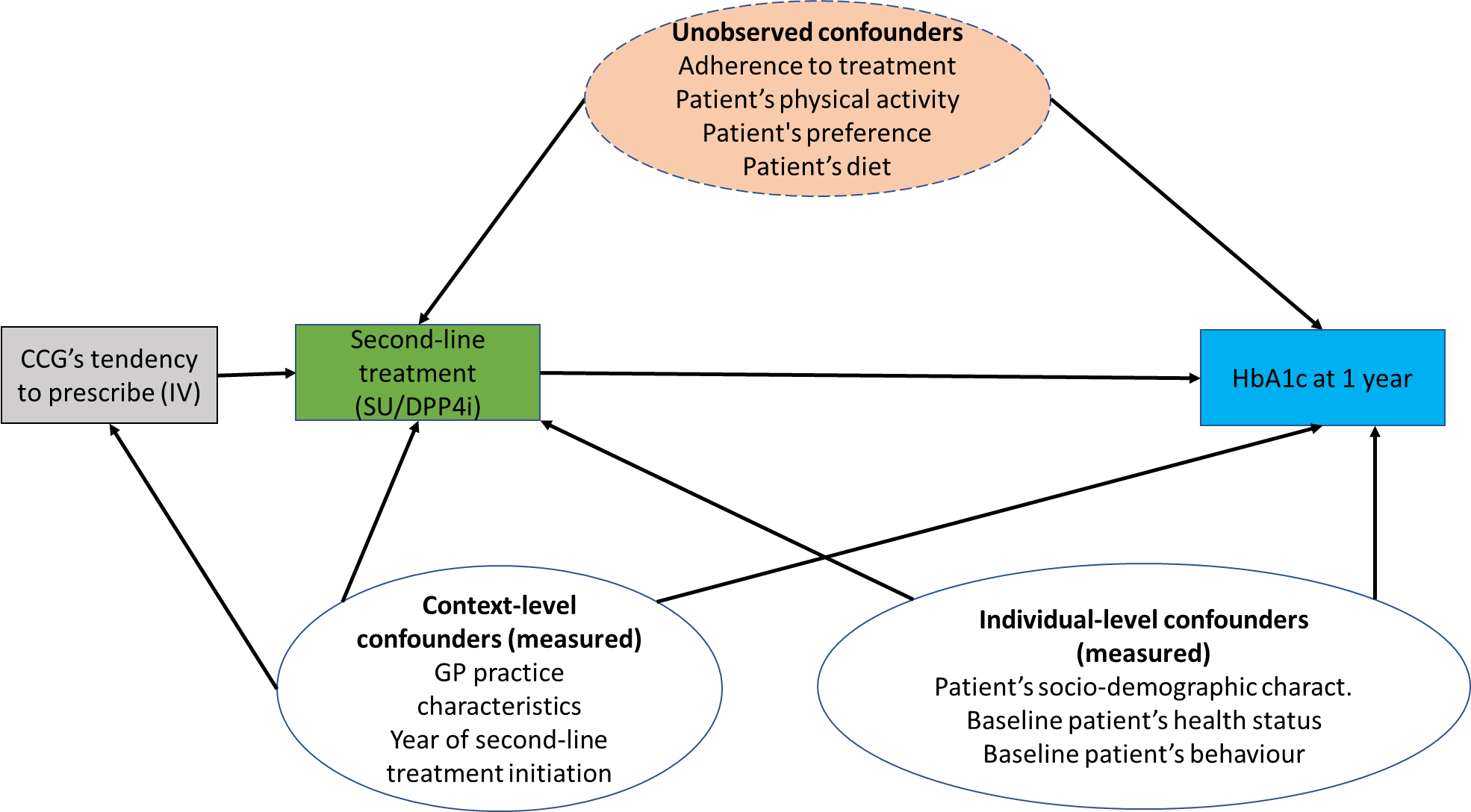
**

**Figure S2. Diagnostic tests for Instrumental Variable**

Mean level of rescaled baseline covariates according to the level of the instrumental variable.

**
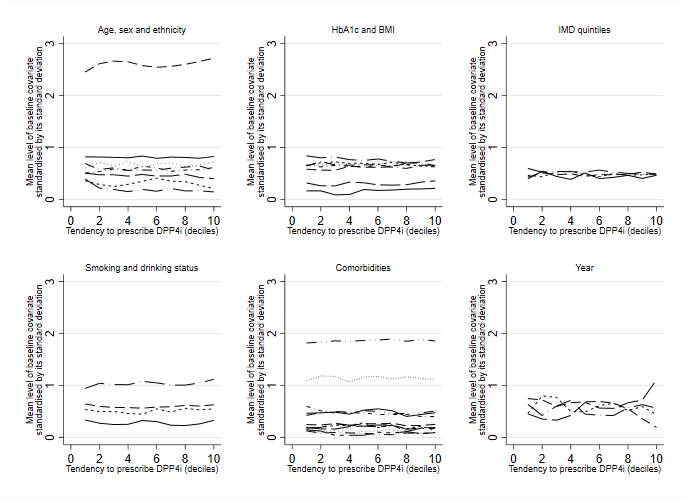
**

**Figure S3:** Flow diagram illustrating the *general population* inclusion and exclusion criteria

**Figure S4:** Distribution of estimated individual treatment effects reported as expected difference (DPP4i-SUs) in change in HbA_1C_ (mmol/mol) between baseline and 1 year for the target population from the LIV approach across pre-specified subgroups.†

1. **
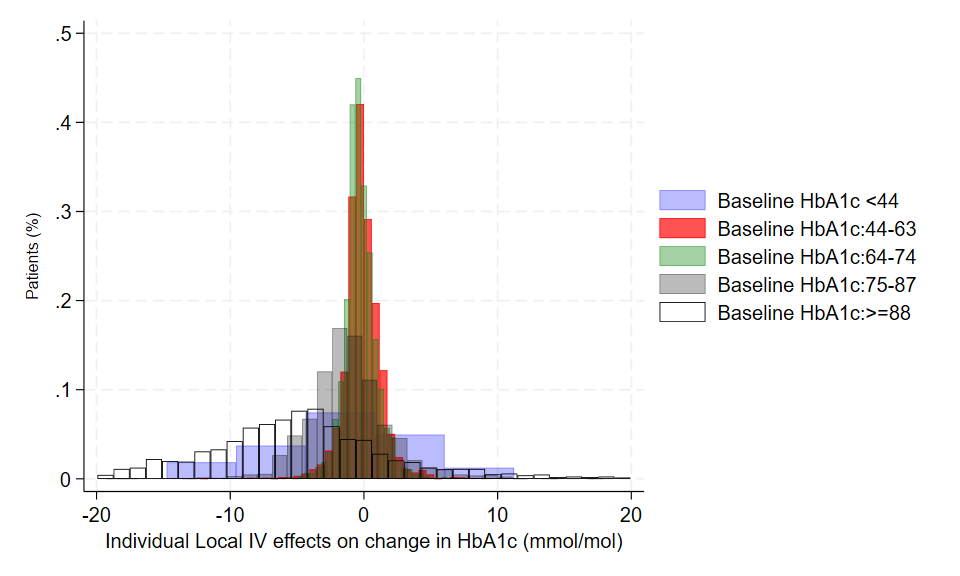
**Across baseline HbA1c subgroups
2. Across age subgroups

**
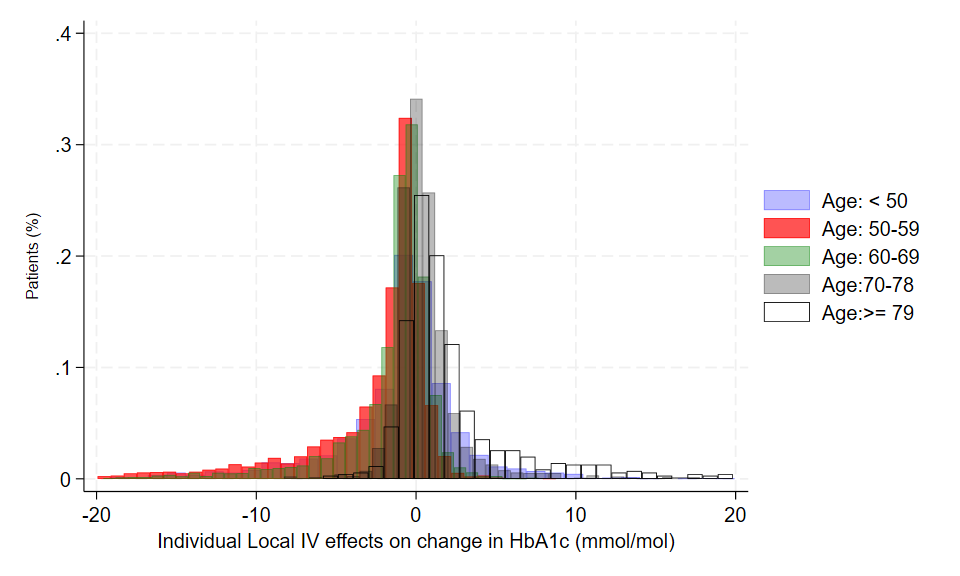
**

† For presentation purposes, 135 individual effects (1% of target population) outside the range (-20,20) were excluded.


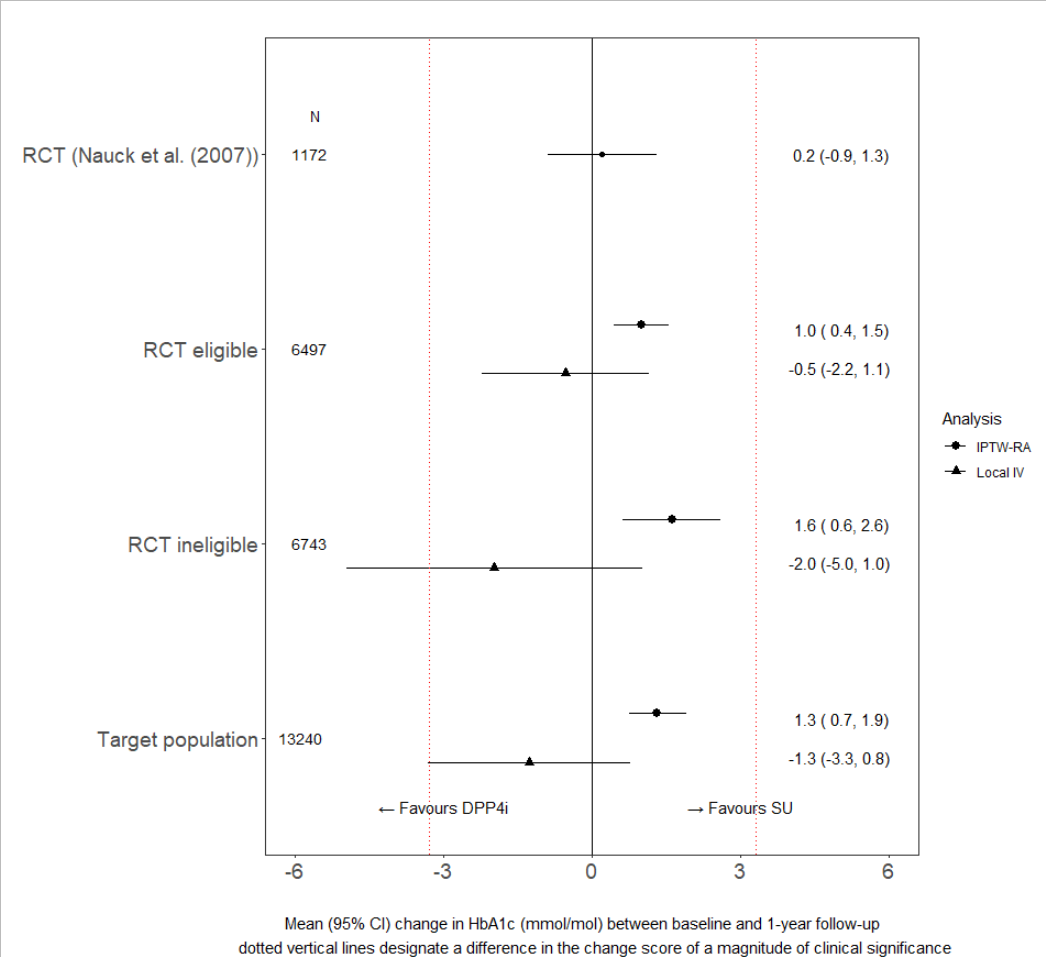
**Figure S5: ATEs from** Nauck et al. (2007), and for the corresponding ‘RCT eligible’ subpopulation, ‘RCT eligible’ and overall target populations from the target trial using the LIV and Inverse probability weighting with regression adjustment (IPTW-RA) approaches. Average Treatment effects (ATEs) reported as difference (DPP4i-SUs) in change in HbA_1C_ (mmol/mol) between baseline and 1 year.

**Figure S6: CATEs** for the ‘RCT eligible’ subpopulation, ‘RCT eligible’ and overall target populations from the target trial using the LIV approach for age and baseline HbA1c subgroups (including subgroup of 32 patients with baseline HbA1c < 44 mmol/mol). Conditional Average Treatment effects (ATEs) reported as difference (DPP4i-SUs) in change in HbA_1C_ (mmol/mol) between baseline and 1 year.

**
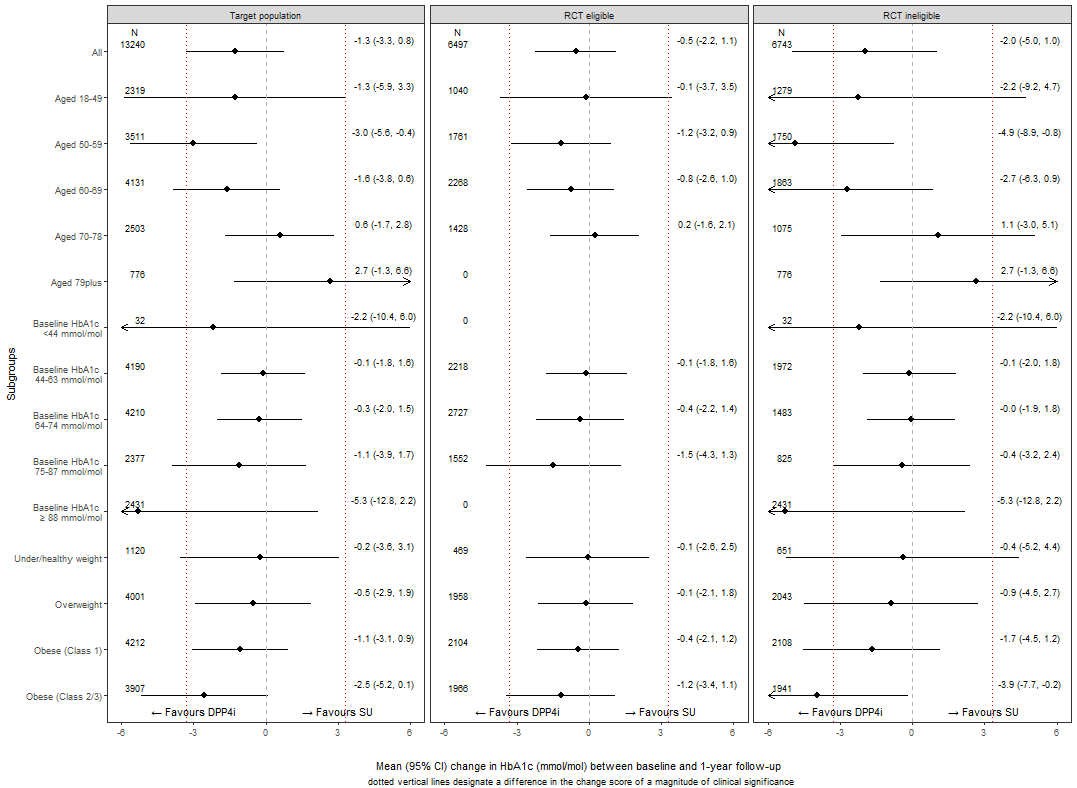
**

**
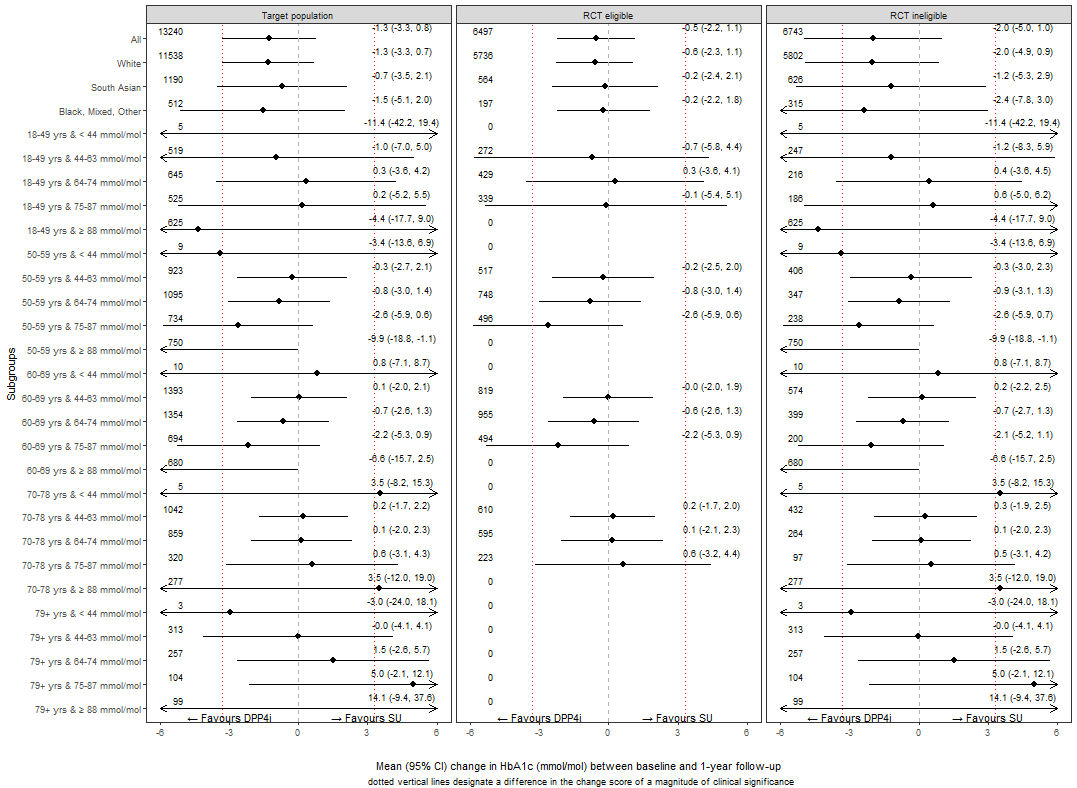
Figure S7: CATEs** for the ‘RCT eligible’ subpopulation, ‘RCT eligible’ and overall target populations from the target trial using the LIV approach for ethnicity and combined age and baseline HbA_1C_ subgroups. Conditional Average Treatment effects (ATEs) reported as difference (DPP4i-SUs) in change in HbA_1C_ (mmol/mol) between baseline and 1 year.

**Figure S8: CATEs** for the ‘RCT eligible’ subpopulation, ‘RCT eligible’ and overall target populations from the target trial using LIV and Inverse probability of treatment weighting with regression adjustment (IPTW-RA) approaches for age and baseline HbA1c subgroups. Conditional Average Treatment effects (ATEs) reported as difference (DPP4i-SUs) in change in HbA_1C_ (mmol/mol) between baseline and 1 year.

**
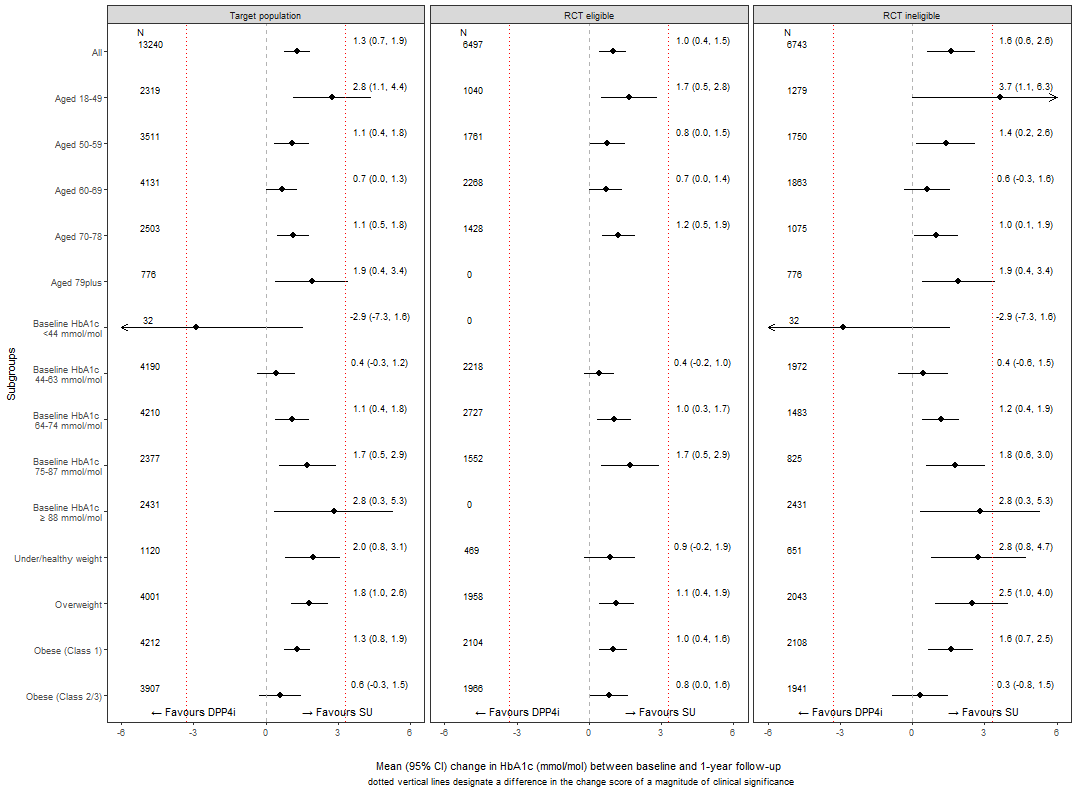
**
